# Effects of Starvation on Deltamethrin Tolerance in Bed Bugs, *Cimex lectularius* L. (Hemiptera: Cimicidae)

**DOI:** 10.3390/insects6010102

**Published:** 2015-01-09

**Authors:** Zachary C. DeVries, William R. Reid, Stephen A. Kells, Arthur G. Appel

**Affiliations:** 1 Department of Entomology and Plant Pathology, 301 Funchess Hall, Auburn University, Auburn, AL 36849, USA; E-Mails: wzr0005@tigermail.auburn.edu (W.R.R.); appelag@auburn.edu (A.G.A.); 2 Department of Entomology, 219 Hodson Hall, 1980 Folwell Ave., University of Minnesota, St. Paul, MN 55108, USA; E-Mail: kells002@umn.edu

**Keywords:** bed bug, deltamethrin, feeding, insecticide, starvation, tolerance

## Abstract

Bed bugs, *Cimex lectularius* L., are a major pest in the urban environment. Their presence often results in physical, psychological, and financial distress of homeowners and apartment dwellers. Although many insecticide bioassays have been performed on this pest, little attention has been paid to bed bug feeding status, which is closely linked to metabolism, molting, and mass. Therefore, we evaluated the toxicity of topically applied deltamethrin on insecticide susceptible adult male bed bugs fed 2 d, 9 d, and 21 d prior to testing. When toxicity was evaluated on a “per-bug” basis, there was no difference between 2 d [LD_50_ = 0.498 (0.316 − 0.692) ng·bug^−1^] and 9 d [LD_50_ = 0.572 (0.436 − 0.724) ng·bug^−1^] starved bugs, while 21 d starved bugs had a significantly lower LD_50_ [0.221 (0.075 − 0.386) ng·bug^−1^]. When toxicity was evaluated in terms of body mass, 9 d starved bugs had the highest LD_50_ values [0.138 (0.102 − 0.176) ng·mg^−1^], followed by 2 d starved bugs [0.095 (0.060 − 0.134) ng·mg^−1^], and then 21 d starved bugs [0.058 (0.019–0.102) ng·mg^−1^]; the LD_50_ values of 2 d and 9 d starved bugs were significantly different from 21 d starved bugs. These results indicate that feeding status plays an important role in the toxicity of deltamethrin. In addition, the lack of differences between 2 d and 9 d starved bugs indicate that the blood meal itself has little impact on tolerance, but rather it is some physiological change following feeding that confers increased tolerance to bed bugs.

## 1. Introduction

Bed bugs, *Cimex lectularius* L., have become a major problem facing society in the twenty-first century. Their presence in residences not only results in bites and skin irritations, but can also cause lasting psychological and financial problems for their victims [1,2,3]. Because of their recent resurgence and the high levels of insecticide resistance encountered in populations globally, bed bugs have become one of the more difficult indoor pest species to manage [4,5,6,7,8,9,10]. Most insecticide bioassays involving bed bugs use bugs starved for some period of time, although starvation times vary widely. Insecticides of various types and formulations have been tested against bed bugs starved for 5 d [11], 8 d [12], and >12 d [13]. Starving bed bugs has been reported to contribute to more consistent mortality measurements [13], however the behavior and physiology of bed bugs make it important to assess tolerance at different times after feeding.

The feeding habits of bed bugs provide a challenge in determining efficacy of insecticides. Blood meals can increase the body mass of bed bugs by 1.5–6.1 times, depending on life stage [14]. Although many studies have investigated mortality to continuous insecticide exposure, these results generally ignore the mass of the insect being tested [11,12,13,15,16]. The per-bug estimate provides numbers that have practical relevance for studies improving control of this pest, but may result in increased within-stadia variability as mass is affected by nutritional status. To understand the effects of feeding on bed bug tolerance to insecticides, toxicity should be evaluated on a mass specific basis (e.g., ng of insecticide per mg of bed bug body mass). Bed bugs also have large fluctuations in metabolic activity affected by molting, feeding and starvation, and they may undergo five or six characteristic stages of metabolic activity within stadia [17]. Elevated metabolic function in response to either molting or feeding could potentially affect the ability of bed bugs to detoxify insecticides, depending on when they are challenged.

Feeding status is also important to consider in terms of insecticide contact. Feeding is important in the growth and development of all organisms, however a single large meal taken in <15 min makes feeding status a particularly important consideration when evaluating exposure to insecticide and the resulting insecticidal efficacy [14]. Many insecticides used against bed bugs require contact with residues. The time available for bed bugs contacting these insecticides can vary tremendously depending on a number of factors including: feeding behavior and nutritional status, host availability, insecticide placement, and the patchiness of the insecticide applications [10,14]. Therefore, understanding how feeding status affects the tolerance of bed bugs to insecticides is important in improving our understanding and management of this pest.

At present, the effect of feeding status on insecticide tolerance in bed bugs has only been evaluated for a short period of time after feeding on a per-bug basis [16]. However, Choe and Campbell [16] did not address the effects of bed bug mass and they did not evaluate starvation times longer than 9 d, times which have been shown to be important both physiologically and behaviorally [17,18]. Therefore, to account for these variables we assessed the effects of bed bug feeding status on the efficacy of topically applied deltamethrin. In this study, LD_50_ values were determined on a field-collected strain of bed bugs considered susceptible to insecticides (J.F. Olson, personal communication [19]). Testing efficacy without the confounding effects of an identifiable resistance mechanism will reveal how feeding status impacts per-bug and mass-specific tolerance to deltamethrin, a common pyrethroid used in bed bug management [20]. To evaluate the interactive effects of metabolism, bed bugs were treated during time periods where substantial changes occur with bed bug metabolic function [17]. The results are interpreted in relation to bed bug behavior and metabolism as well as other hematophagous insects and other dose-response studies.

## 2. Materials and Methods

### 2.1. Experimental Animals

An insecticide susceptible laboratory strain of bed bugs originally obtained from i2L Research, Inc. (Baltimore, MD, USA) was reared and maintained at the University of Minnesota, Twin Cities, MN following the procedures of Olson *et al.* [18]. Bed bugs were fed weekly on expired human blood obtained from the American Red Cross (St. Paul, MN, USA) using an artificial feeding system as described by Montes *et al.* [21]. Bed bug colonies were reared in 0.5 L glass jars at 23 ± 2 °C, 55% ± 5% RH and a photoperiod of 14:10 (L:D) h. Adult male bed bugs were shipped to Auburn, AL, USA overnight immediately following feeding. Bed bugs were shipped in groups of 100 in plastic cylinders (0.5 L) with mesh tops to allow for ventilation and filter paper for harborage.

### 2.2. Topical Application Experiments

Groups of 7–10 bed bugs were tested together to determine the effects of feeding status on bed bug tolerance to deltamethrin. Bed bugs fed the same diet were tested at one of three times after feeding: 2 d, 9 d, or 21 d. These three times are important both in terms of the bed bugs life history and metabolism. Two days represents the highest metabolic rate observed in adult bed bugs, 9 d represents a stable intermediate metabolic rate observed in bed bugs along with a time bed bugs will generally be in search of another blood meal, and 21 d represents a depressed metabolic rate during starvation [17,22]. Bed bugs were removed from the 0.5 L plastic cylinder and placed into plastic Petri dishes (diameter = 4 cm; Falcon Plastics, Brookings, SD, USA) with a Whatman no. 1 filter paper substrate (diameter = 4 cm; GE Healthcare Life Sciences, Little Chalfont, Buckinghamshire, UK) covering the base of the Petri dish and another half-piece of Whatman no. 1 filter paper folded in an accordion design, which served as harborage for the bed bugs. The Petri dish was weighed both before and after placing bed bugs inside to determine the mass of the bed bugs in groups, and then divided by the number of bed bugs to determine the average individual bed bug mass. Massing bed bugs in groups minimized handling time and avoided any possible negative effects from excessive handling. After determining mass, bed bugs in the Petri dishes were placed on ice and treated topically with 0.5 µL of technical grade deltamethrin (99.9% a.i., FMC Corporation, Princeton, NJ, USA) diluted in acetone using a 10 µL glass syringe held in a repeating micro-pipette (Hamilton Company, Reno, NV, USA). Doses ranged from 0 ng (control, acetone only) to 10 ng (0, 0.5, 1, 2, 5, 10 ng). Mortality was assessed after 24 h and was determined by the inability of bed bugs to move 1 body length in 5 s while being agitated with a probe one time every second. This measure was used because some bed bugs exhibited irreversible moribundity, but were still observed twitching >14 d after treatment. At least 4 replicates (with 7–10 bugs each) were made for each feeding status-dose combination tested.

### 2.3. Data Analysis

Lethal doses were determined using probit analysis in PoloPlus (LeOra Software Company, Petaluma, CA, USA) to generate LD_50_ values for each feeding status (2 d, 9 d, 21 d starved) [23]. These values were determined on a per-bug and a per-body-mass basis. LD_50_ values were compared among post-feeding periods; significant differences were determined using the lethal dose ratio test, where two LD_50_ values are not significantly different if the 95% confidence interval of the ratio includes 1 [23]. In addition, the slopes of the probit regression were also compared among post-feeding periods using PoloPlus, with significant differences based on non-overlapping 95% CIs of the coefficients.

## 3. Results

### 3.1. Mortality-Per Bug

Feeding status significantly affected the efficacy of topically applied deltamethrin when assessed on a per-bug basis. The LD_50_ of bed bugs, which were fed either 2 d (0.498 ng·bug^−1^) or 9 d (0.572 ng·bug^−1^) before testing, showed no significant difference (Ratio = 0.870 (0.558 − 1.356), Table 1). However, bed bugs fed 21 d before testing had a significantly lower LD_50_ (0.221 ng·bug^−1^) than 2 d starved bed bugs (Ratio = 2.254 (1.424 − 3.568), Table 1) and 9 d starved bed bugs (Ratio = 2.590 (1.801 − 3.725), Table 1). The greatest difference existed between 9 d starved bed bugs and 21 d starved bed bugs, representing a 2.59 times greater LD_50_ for the 9 d starved bed bugs.

The slopes of the dose-response lines for the “per-bug” doses were compared among different feeding statuses (Table 1). Bed bugs fed 2 d (1.635) before testing showed the lowest slope and were significantly different from bed bugs fed 9 d (3.020) and bed bugs fed 21 d (2.569) before testing.

### 3.2. Mortality-Mass Specific

Feeding status significantly affected the efficacy of topically applied deltamethrin when assessed on a mass specific basis (ng·mg^−1^). The LD_50_ of bed bugs fed 9 d before testing showed the highest tolerance (0.138 ng·mg^−1^) and was significantly greater than bed bugs fed 21 d before testing (0.058 ng·mg^−1^) (Ratio = 2.365 (1.624 − 3.444); Table 1). Bed bugs fed 2 d before testing (0.095 ng·mg^−1^) showed the second highest tolerance, although they were only significantly different from 21 d starved bed bugs (Ratio = 1.640 (1.028 − 2.615), Table 1) and not significantly different from 9 d starved bed bugs (Ratio = 0.693 (0.438 − 1.098), Table 1).

The slopes of the dose-response lines for the mass-specific doses were compared among different feeding statuses (Table 1). Bed bugs fed 2 d before testing had a significantly lower slope (1.602) than the other two feeding status. Bed bugs fed either 9 d (2.869) or 21 d (2.577) before testing were not significantly different from each other.

**Table 1:** Per-bug and mass-specific mortality results for bed bugs tested at three different post-feeding times. Sample size (*n*) is reported; however because bed bugs were massed in groups the sample size for mean mass is lower and is indicated in parentheses. Slopes followed by different letters are significantly different with no overlap of the standard errors (SE). LD_50_ values followed by different letters are significantly different according to the lethal dose ratio test.

			Per Bug Mortality			Mass-Specific Mortality		
Post-Feeding Time	*n*	Mean Mass (±SE) (mg)	Slope (±SE)	LD_50_ (95% CI) (ng·bug^−1^)	χ^2^ (*d.f.*)	Slope (±SE)	LD_50_ (95% CI) (ng·mg^−1^)	χ^2^ (*d.f.*)
2 d	150 (24)	5.25 (±0.09)	1.635 (±0.280) ^a^	0.498 (0.316 − 0.692) ^a^	0.852 (3)	1.602 (±0.273) ^a^	0.095 (0.060 − 0.134) ^a^	0.637 (3)
9 d	120 (20)	4.26 (±0.10)	3.020 (±0.518) ^b^	0.572 (0.436 − 0.724) ^a^	0.201 (2)	2.869 (±0.501) ^b^	0.138 (0.102 − 0.176) ^a^	0.380 (2)
21 d	172 (29)	3.80 (±0.06)	2.569 (±0.370) ^b^	0.221 (0.075 − 0.386) ^b^	2.119 (2)	2.577 (±0.372) ^b^	0.058 (0.019 − 0.102) ^b^	2.177 (2)

## 4. Discussion

This study demonstrates that bed bug feeding status plays a significant role in the toxicity of insecticides when topically applied to bed bugs. When dose per bug is considered, bed bugs recently fed (2 d) or starved for 9 d showed similar response to the same dose of insecticides. However, as starvation increased, we observed decreased tolerance, with 21 d starved bed bugs having significantly lower LD_50_ values (Table 1). This response indicates that although bed bug mass starts to decline immediately following feeding, their ability to tolerate insecticides does not change until after 9 d post-feeding. This was surprising, because previous studies involving mosquitoes demonstrated that a blood meal only increased insecticidal tolerance for two to four days [24,25]. One possibility for this difference is the time required for bed bugs to fully digest a blood meal. *Culex pipiens* L. only requires 70 h to fully digest a blood meal, while bed bugs typically take up to a week at 25 °C before feeding, suggesting a much longer blood processing time [2,26]. Another possibility is the difference in sexes between studies. We evaluated only adult males, while the previous studies on mosquitoes were forced to evaluate only females because they are the only life stage that consumes a blood meal [24,25]. Feldlaufer *et al.* [12] reported no difference in the toxicity of insecticides between males and females, however they only measured the effects of continuous exposure. In addition, Choe and Campbell [16] found a significant difference in the tolerance of 1 d and 9 d starved bed bugs when continually exposed to dried residues of deltamethrin, but no differences at these times when topically applied. Therefore, further studies are needed to evaluate differences between topical application and continual exposure, as well as differences in topical LD_50_ values between sexes.

Mass specific results revealed bed bugs starved for 9 d had the greatest tolerance to deltamethrin (Table 1), followed by those starved 2 d, and finally those starved 21 d. Significant differences were only detected between those starved 2 d or 9 d *versus* those starved 21 d (Table 1). When evaluated alongside the per-bug measurements, both measures indicate that a blood meal itself does little to directly increase tolerance. A similar result was observed for an insecticide-susceptible strain of *Anopheles funestus* Giles, where taking a blood meal 24 h prior to insecticide (permethrin) treatment resulted in no significant change in tolerance [27]. However, Spillings *et al.* [27] also found that an insecticide-resistant strain of *An. funestus* was more tolerant to permethrin exposure after feeding. Spillings *et al.* [27] hypothesized that the increased permethrin tolerance observed in the insecticide-resistant strain following a blood meal was due to the relatively higher gene expression levels of cytochrome P450 genes that were up-regulated in response to blood feeding. Although not tested in this study, we would hypothesize that the observed shift in tolerance is due to fluctuations in the quantities and activities of detoxification enzymes. Decreased penetration could also play a role since the morphology of the bed bug changes during starvation, especially from 2 d after feeding until 9 d after feeding when the cuticle becomes less stretched (thicker) as the bed bug returns to its flat state [14,24,28]. Mosquitoes have been shown to increase cytochrome P450 and epoxidation activity following a blood meal, although specific mechanisms are not fully understood [24]. Other blood feeding arthropods, including *Rhodnius prolixus* Staͦl, also upregulate detoxification gene expression following a blood meal, likely as a response to the large quantities of hemoglobin that could otherwise be toxic [29,30]. It is possible that there is some cross activity between enzymes involved in blood digestion and those involved in insecticide detoxification as observed in insecticide-resistant *A. funestus*, meaning the same enzymes used to digest blood meals may also be used to metabolize insecticides [27]. Insecticide detoxification genes are expressed predominantly in the cuticular tissue (epidermis) of insecticide-resistant bed bugs [31], and since there is a change in the morphology of the bed bug cuticle during starvation [28], there may be an interaction between cuticular morphology and detoxification enzyme activity, but additional studies are required to determine the interactions with metabolism in this system.

The lower slope for the recently fed (2 d) bed bugs (both per-bug and mass-specific) indicates they have a larger range of digestion statuses and therefore enzymatic function than either the 9 d or 21 d starved bed bugs. This is not surprising because blood meal digestion is likely occurring at different rates among recently fed individuals, while at 9 d and 21 d post-feeding there appears to be more uniformity, as the majority of this blood meal would be fully processed.

Blood feeding is an important consideration when conducting insecticide bioassays with hematophagous insects. The World Health Organization has standards for mosquito testing (non-blood fed, 3–5 d post emergence), however no such standard exist for bed bugs [32]. Numerous studies have tested bed bugs at various times post feeding, ranging from 5 d to >12 d [11,12,13]. Difference in feeding status among tests may confound our understanding of the effects of various insecticides, which is critical as we continue to search for the most effective management strategy. Because feeding could potentially alter any number of physiological and behavioral factors, a standard of testing should be developed to compare results among research laboratories.

Additionally, other forms of control should also be evaluated at various post-feeding times to better understand how a blood meal may impact bed bug management. It is not uncommon to find bed bugs ranging from fully engorged to starved within the same home, especially if the infestation is large and dispersed. Thus, a complete understanding of the effects of feeding status on various management practices is important to improving bed bug control.

## 5. Conclusions

In conclusion, periods of prolonged starvation (21 d) significantly lowered bed bug tolerance to deltamethrin, both on a per-bug and a mass-specific basis, when compared to bed bugs that had recently fed (2 d). However, bed bugs starved for shorter periods (9 d) did not differ in deltamethrin tolerance from recently fed bed bugs (2 d). These findings suggest that the blood meal itself does not increase tolerance, but rather physiological changes following feeding confer greater tolerance to 2 d and 9 d starved bed bugs compared with those starved 21 d. Although the mechanisms remain unknown, we suspect that general decreased enzymatic activity during starvation is responsible. Future studies should aim to better understand the mechanisms affecting insecticide tolerance during starvation and should also evaluate different chemical classes to determine if feeding status has any impact on insecticides with different modes of action.
